# Serum Sodium Profile of Congestive Heart Failure Patients and its Impact on Their Outcome at Discharge

**DOI:** 10.7759/cureus.5462

**Published:** 2019-08-22

**Authors:** Tuba Mahmood, Kuldeep Raj, Moiz Ehtesham, Joty Bhimani, Shah Jabeen, Amber Tahir

**Affiliations:** 1 Cardiology, Quaid E Azam Medical College, Bahawalpur, PAK; 2 Internal Medicine, Jinnah Sindh Medical University, Karachi, PAK; 3 Internal Medicine, Dow International Medical College, Dow University of Health Sciences, Karachi, PAK; 4 Internal Medicine, Liaquat University of Medical and Health Sciences, Karachi, PAK; 5 Physiology, Liaquat College of Medicine and Dentistry, Karachi, PAK; 6 Internal Medicine, Dow University of Health Sciences, Karachi, PAK

**Keywords:** hyponatremia, dilutional hyponatremia, congestive heart failure, predictors of mortality, in-hospital outcomes

## Abstract

Introduction

Patients with congestive heart failure (CHF) readily present with electrolyte imbalance which commonly includes deficiencies of sodium, potassium, and magnesium. Hyponatremia occurs in advanced stages of CHF and is associated with adverse disease outcome-longer hospital stay, severity of CHF, and increased risk of mortality.

Methods

In this observational, single-center, prospective, case-control study adult patients admitted with clinical diagnosis of CHF were included after informed consent. Their demographic, clinical, and biochemical profile was attained. Patients with low serum sodium (hyponatremia) were grouped as "cases" and patients with normal serum sodium profile (normonatremia) were grouped as "controls". Factors associated with both groups and their hospital outcome were compared. SPSS for Windows version 16 (SPSS Inc., Chicago, IL, USA) was utilized.

Results

Hyponatremia (serum sodium <135 mmol/L) was present in 58/189 (30.7%) patients admitted with CHF. Younger patients with non-ischemic CHF, and history of previous diagnosis, treatment, and hospitalization due to CHF were more likely to be affected. Diabetic nephropathy, chronic kidney disease, salt-restricted diet, drugs including furosemide, spironolactone, and angiotensin-converting enzyme inhibitors, low serum potassium, and reduced GFR were also related to hyponatremia. Hyponatremic CHF patients showed adverse hospital outcome on all parameters including higher death rate (12% vs. 0.8%), longer duration of hospital day, and deranged blood pressures and severe CHF at the time of discharge.

Conclusion

Hyponatremic CHF patients are associated with prolonged hospital stay, more severe form of CHF, and deranged blood pressures. Overall, hyponatremia is an indirect clinical indicator of circulatory dysfunction and should guide a clinician for closer observation as outcomes could be poor. These patients also have higher in-hospital mortality risk.

## Introduction

Congestive heart failure (CHF) is a clinical syndrome with functional as well as structural disruptions in the myocardium. In earlier stages, ventricular filling is preserved, however, as the disease progresses, there is impairment of ventricular filling (reduced ejection fraction). Myocardial ischemia remains the most common cause of CHF. Other causes include dysfunction of the pericardium, myocardium, endocardium, heart valves, or great vessels alone or in combination [1].

Patients with CHF readily present with electrolyte imbalance both in the outpatient and the inpatient department. This may be due to activation of neurohumoral mechanism due to heart failure state (stimulation of the renin-angiotensin-aldosterone system, sympathoadrenergic stimulation) or it may occur as an unwanted effect of certain drugs such as diuretics, cardiac glycosides, and angiotensin-converting enzyme inhibitors (ACEi). The common electrolyte imbalance seen in these patients include deficiencies of sodium, potassium, and magnesium. Deficiencies of magnesium and potassium pose a serious risk of cardiac arrhythmias [2]. Hyponatremia occurs in advanced stages of CHF because of impaired urinary water excretion. There is excess of water in relation to potassium and sodium concentrations, hence, this type of hyponatremia is called dilutional or hypotonic. Other causes of hypoantremia in advanced CHF include increased production of antidiuretic hormone (water diuresis effect), use of potent diuretic drugs, and severe salt restriction [3].

Reduced serum sodium concentrations have been associated with adverse disease outcome-longer hospital stay, severity of CHF on New York Heart Association (NYHA) functional class, predisposition to fall and fractures, and increased risk of mortality [3-4]. The aim of this study is to determine the frequency of hyponatremia in hospitalized patients of CHF and evaluate its impact on their hospital outcome. Understanding the importance of sodium profile will help clinicians observe and evaluate patients with low sodium more closely to control poor outcome.

## Materials and methods

This observational, prospective, and case-control study was conducted in Civil Hospital located in Larkana, Pakistan. The study was conducted in the department of internal medicine from January to December 2018. The study was approved by the Institutional Review Board.

 In this study, patients of age ≥ 18 years, of both genders, admitted with the clinical diagnosis of CHF of all etiologies were included after attaining informed consent. CHF was diagnosed according to Framingham Criteria. Major criteria include paroxysmal nocturnal dyspnea or orthopnea, neck-vein distention, rales, cardiomegaly, acute pulmonary edema, S3 gallop, increased venous pressure >16 cm of water, circulation time ≥25 seconds, and hepatojugular reflux. Minor criteria include ankle edema, night cough, dyspnea on exertion, hepatomegaly, pleural effusion, vital capacity ↓ ⅓ from maximum, and tachycardia (rate of ≥ 120/min). Major or minor criterion includes weight loss ≥4.5 kg in 5 days in response to treatment. Two major criteria or one major and two minor criteria are needed for diagnosis of CHF [5].

A semi-structured questionnaire was constructed for data collection. First section of the questionnaire included demographic, clinical, and biochemical characteristics. Demographic characteristics comprised of gender and age. Past medical history included co-morbidities, previous hospital admissions, and drug history. Thorough clinical examination was done for diagnosis of CHF. Severity of CHF was assessed using NYHA functional class [6].

Relevant biochemical data comprised of serum creatinine, blood urea nitrogen, serum sodium, and serum potassium. Second part of the questionnaire was filled at the time of discharge to assess hospital outcome. It included alive/dead status, duration of hospital stay, blood pressure at the time of discharge, and CHF severity based on NYHA Classification.

For the purpose of analysis, patients of serum sodium level (at the time of admission) <135 mmol/L (hyponatremia) were categorized as "cases" and patients with serum sodium 135-145 mmol/L (normonatremia) were grouped as "controls". Glomerular filtration rate (GFR) was calculated for all patients [3,7]. All clinical and laboratory parameters included in this study were a part of routine patient evaluation. Patients with serum sodium >145 mmol/L were not included in the study. Hence, the study did not request, recommend, or burden the patients/physicians with any additional investigation.

Data were assessed for cleanliness, completeness, and consistency. It was then entered for analysis in SPSS for Windows version 16 (SPSS Inc., Chicago, IL, USA). Continuous variables were presented as mean and standard deviation (SD). Categorical variables were presented as frequencies and percentages. For the purpose of analysis, data were divided into two categories based on serum sodium concentration (“≥135 mmol/L” and “<135 mmol/L”). Independent sample t-test was applied to find the correlation between continuous variables. Chi-square test was applied to correlate categorical variables. Risk was presented as odds ratio (ORs) and confidence interval (CI). P value ≤0.05 was taken as significant.

## Results

In this study, 189 patients with CHF were included. There were more women than men (56.1% vs. 43.9%). The mean age of the study sample was 53 ± 7 years (range: 47-68 years). There were 72 (38.1%) referred from other smaller cities, 38 (20.1%) belonged to the outskirts of the city, and 98 (51.8%) patients were illiterate or only had primary education.

In 46% patients, CHF was due to ischemia and in the remaining 54% patients, causes included hypertension (n=55; 29.1%), valvular disorders (n=28; 14.8%), dilated/restrictive cardiomyopathies (n=12; 6.3%), and others (n=6; 3.2%) had been previously diagnosed in 36%, treated in 30%, and 26% had been previously hospitalized due to HF. At the time of admission, all patients had dyspnea on exertion, 93.1% (n=176) had dyspnea on rest, 88.9% (n=168) had orthopnea, and 87.8% (n=166) had paroxysmal nocturnal dyspnea. A large majority of 89.9% (n=170) presented with Class IV NYHA and remaining with Class III HF. Mean serum potassium level of the study sample at the time of presentation was 4.56 ± 1.03 mmol/L, GFR was 96.5 ± 48.8 (mL/minute/1.73 m2), and serum sodium level was 132.25 ± 6.14 mmol/L/. There were 131 (69.3%) patients with serum sodium ≥135 mmol/L (normonatremia) and 58 (30.7%) patients with serum sodium <135 mmol/L (hyponatremia).

Demographic and disease-related characteristics of the study samples categorized according to their serum sodium concentrations are shown in table 1. As evaluated from table 1, younger age was associated with hyponatremia (p=0.02). When CHF-related characteristics were assessed, it was seen that non-ischemic causes of CHF, previous diagnosis (OR: 7.22; CI: 3.64, 14.34), treatment (OR: 5.89; CI: 2.97, 11.65), and hospitalization due to CHF (OR: 4.89; CI: 2.44, 9.8) were all related to hyponatremia (p <0.000). In comorbidity status, only diabetic nephropathy (OR: 4.52; CI: 1.96, 10.45; p=0.000) and chronic kidney disease (OR: 4.63; CI: 1.48, 14.5; p=0.004) were related to hyponatremia in HF patients. Other important characteristics included salt-restricted diet, use of furosemide, spironolactone, and ACEi, low serum potassium and reduced GFR. The only protective factor for hyponatremia in HF patients was additional dietary intake of sodium (OR: 0.37; CI: 0.19, 0.7; p=0.002) (table 1).

**Table 1:** Demographic and Clinical Factors Associated With Hyponatremia in Patients Admitted with Congestive Heart Failure (N=189) ACE: Angiotensin-converting enzyme; GFR: Glomerular filtration rate; HF: Heart failure; SD: Standard deviation * All results indicate correlation between “Serum sodium ≥135 mmol/L” and “Serum sodium <135 mmol/L” groups only

Patient Characteristics	Total patients	Serum sodium ≥135 mmol/L	Serum sodium <135 mmol/L	Odds Ratio*	Confidence Interval*	P value*
Gender, n (%)						
Male	83 (43.9%)	53 (63.8%)	30 (36.2%)	1.58	0.85, 2.94	0.15
Female	106 (56.1%)	78 (73.6%)	28 (26.4%)			
Age in years, mean ± SD	53 ± 7	51 ± 2	50 ± 4	---	---	0.02
Causes of HF, n (%)						
Ischemic causes	88 (46.6%)	68 (77.3%)	20 (22.7%)	0.57	0.3, 1.09	0.02
Non-ischemic causes	101 (53.4%)	63 (62.4%)	38 (37.6%)			
Previous diagnosis of HF, n (%)						
Yes	68 (36.0%)	29 (42.6%)	39 (57.4%)	7.22	3.64, 14.34	<0.000
No	121 (64.0%)	102 (82.3%)	19 (15.7%)			
Previous hospitalization due to HF, n (%)						
Yes	49 (25.9%)	21 (42.8%)	28 (57.1%)	4.89	2.44, 9.8	<0.000
No	140 (74.1%)	110 (78.6%)	30 (21.4%)			
Prior treatment for HF, n (%)						
Yes	57 (30.2%)	24 (42.1%)	33 (57.9%)	5.89	2.97, 11.65	<0.000
No	132 (69.8%)	107 (81.1%)	25 (18.9%)			
Diabetes mellitus, n (%)						
Yes	67 (35.4%)	43 (64.2%)	24 (35.8%)	1.44	0.76, 2.73	0.25
No	122 (64.5%)	88 (72.1%)	34 (27.8%)			
Diabetic nephropathy, n (%)						
Yes	28 (14.8%)	11 (39.3%)	17 (60.7%)	4.52	1.96, 10.45	0.000
No	161 (85.2%)	120 (74.5%)	41 (25.5%)			
Chronic kidney disease, n (%)						
Yes	14 (7.4%)	5 (35.7%)	9 (64.3%)	4.63	1.48, 14.5	0.004
No	175 (92.6%)	126 (72.0%)	49 (28.0%)			
Hypertension, n (%)						
Yes	43 (22.8%)	26 (60.5%)	17 (39.5%)	1.67	0.82, 3.41	0.15
No	146 (77.2%)	105 (71.9%)	41 (28.1%)			
Additional salt use, n (%)						
Yes	97 (51.3%)	77 (79.4%)	20 (20.6%)	0.37	0.19, 0.7	0.002
No	92 (48.7%)	54 (58.7%)	38 (41.3%)			
Salt restricted diet, n (%)						
Yes	48 (25.4%)	17 (35.4%)	31 (64.6%)	7.7	3.73, 15.9	<0.000
No	141 (74.6%)	114 (80.9%)	27 (19.1%)			
Furosemide, n (%)						
Yes	38 (20.2%)	16 (42.1%)	22 (57.9%)	4.39	2.09, 9.25	0.000
No	151 (79.8%)	115 (76.2%)	36 (23.8%)			
Hydrocholorthiazide, n (%)						
Yes	11 (5.8%)	8 (72.7%)	3 (27.3%)	0.84	0.21, 3.28	0.800
No	178 (94.2%)	123 (69.1%)	55 (30.9%)			
Spironolactone, n (%)						
Yes	21 (11.1%)	8 (38.1%)	13 (61.9%)	4.44	1.73, 11.42	0.001
No	168 (88.9%)	123 (73.2%)	45 (26.8%)			
ACE inhibitors, n (%)						
Yes	36 (19.1%)	20 (55.6%)	16 (44.4%)	2.11	1.0, 4.46	0.04
No	153 (80.9%)	111 (72.5%)	42 (27.5%)			
Serum potassium (mmol/L), mean ± SD	4.56 ± 1.03	4.63 ± 1.58	4.02 ± 0.87	---	---	0.006
GFR (mL / minute / 1.73 m^2^), mean ± SD	96.5 ± 48.8	92.9 ± 39.3	80.3 ± 30.8	---	---	0.03

Patients with hyponatremia showed adverse hospital outcome on all parameters as shown in table 2. There was 12% death in hyponatremia group as compared to only 0.8% in normonatremia group (p=0.000). The risk of death was 17 times higher in this group (OR: 17.84; CI: 2.14, 148.68). Hyponatremic patients had a four-times of being discharged at severe CHF (class III-IV). These patients also had a longer duration of hospital stay and deranged blood pressures (BPs) at the time of discharge as shown in table 2.

**Table 2:** Comparison of Hospital Outcome of Congestive Heart Failure Patients With and Without Hyponatremia (N=189) NYHA: New York Heart Association; SD: Standard deviation

Hospital Outcome	Serum sodium ≥135 mmol/L	Serum sodium <135 mmol/L	Odds Ratio	Confidence Interval	P value
At the time of discharge, n (%)					
Alive	130 (99.2%)	51 (87.9%)	17.84	2.14, 148.68	0.000
Dead	1 (0.8%)	7 (12.1%)			
Duration of hospital stay in days, mean ± SD					
	9 ± 3	11 ± 5	---	---	0.000
NYHA class at the time of discharge, n (%)					
Class I-II	108 (82.4%)	31 (53.5%)	4.09	2.06, 8.11	0.000
Class III-IV	23 (17.6%)	27 (46.5%)			
Blood pressure at the time of discharge in mmHg, mean ± SD					
Systolic	121 ± 8	111 ± 10	---	---	<0.000
Diastolic	77 ± 5	68 ± 8	---	---	<0.000

## Discussion

Being one of the most common electrolyte imbalance, hyponatremia is readily suspected in hospitalized patients with cardiological, neurological, and/or endocrinological diseases. CHF may be chronic, acute, or acute on chronic. Younger patients, with non-ischemic CHF, and history of previous diagnosis, treatment, and hospitalization due to CHF should be considered high risk for hyponatremia. Diabetic nephropathy, chronic kidney disease, salt-restricted diet, use of drugs including furosemide, spironolactone and ACEi, low serum potassium, and reduced GFR are also related to hyponatremia. These patients have adverse outcome including higher mortality rate and longer duration of the hospital day.

Although this study is crucial in highlighting the importance of hyponatremia in CHF patients, it has its limitations too. It was a single-center study. Only at-discharge outcomes were analyzed and patients couldn’t be followed up to evaluate the long-term impact. The study also included patients with adverse renal function (diabetic nephropathy and chronic kidney disease patients) which may have caused biasness in the results. Most patients belonged to smaller cities or outskirts and were of low education status, hence, the results cannot be generalized to the entire population.

CHF patients have been previously evaluated for hyponatremia in Pakistan [8-10]. Hyponatremia has been reported in 28%-35% patients admitted with CHF [8-10]. Only one study evaluated hyponatremic patients for risk factors, however, they did not find any statistically significant factor. The three-month mortality rate was higher in hyponatremia group (23% vs. 11%; p=0.02). Three-month readmission rates were also higher in this group, however, the differences were not significant [8].

Hyponatremia was associated with 31% of patients presenting with CHF without any other clause. However, this finding must be interpreted closely as other variables such as medications, cause of the CHF, presence of diabetes, chronic and disease might alter the levels of sodium and hence the interpretation of this study. ​​​​The incidence is slightly higher than that reported in the global data. Japanese Acute Decompensated Heart Failure Syndromes (ATTEND) Registry reported 11.6% hyponatremics, Korean heart failure Registry (KorHF) reported 18%, Organized Program to Initiate Lifesaving Treatment in Hospitalized Patients with Heart Failure (OPTIMIZE-HF) reported 19.7%, Evaluation Study of Congestive Heart Failure and Pulmonary Artery Catheter Effectiveness (ESCAPE) trial reported 24%, and Prospective Trial of Intravenous Milrinone for Exacerbations of Chronic Heart Failure (OPTIME-CHF) trial reported 27% hyponatremia [11-15].

ATTEND Registry reported that there were fewer men in the hyponatremia group, they had more previous HF-related hospitalizations and higher rates of all-cause mortality as well as cardiac mortality [11]. Similarly, in the ESCAPE trial, although, hemodynamic and clinical improvement was comparable in both hyponatremia and normonatremia group, hyponatremia was an independent predictor of all-cause mortality and HF-related rehospitalization [14]. KorHF also concluded hyponatremia as an independent predictor of mortality in CHF [12]. In a meta-analysis of 22 studies involving 14,766 patients, patients with hyponatremia had higher NYHA class and lower BP. Mortality risk increases linearly with serum sodium concentration <140 mmol/L. Hyponatremia was an independent predictor of mortality in HF patients regardless of preserved or reduced ejection fraction [16]. In another report with 15% incidence of hyponatremia in acute decompensated HF (ADHF), hyponatremic patients were significantly more globally comorbid, had significantly higher levels of blood glucose, urea, creatinine, and potassium, and lower BP as compared to the normonatremic group. The findings are slightly different from our study as we reported lower serum potassium and lower GFR in these patients. They also did not find any significant association of diuretic or ACEi use with hyponatremia, unlike our results. Rate of readmission was not significantly associated with hyponatremia in their report; however, higher risk of mortality was - as in our analysis [4]. In another study, the cardiac prognosis was poor in hospitalized ADHF patients who were normonatremic at the time of admission and progressed to hyponatremia within the hospital [17].

At the time of discharge, the hyponatremic CHF patients in our study had significantly lower blood pressures as compared to the normonatremic group. These findings are strengthened by another study where hyponatremic patients were discharged at lower blood pressures. More hyponatremic patients belonged to NYHA class III-IV in their study and had a longer duration of hospital stay. The mortality rate was significantly less in the normonatremic group (0.9% vs. 11.3%; p=0.008) [18]. A similar pattern of adverse hospital outcome was seen in our study.

## Conclusions

CHF patients should be readily monitored for electrolyte imbalance. These patients are frequently seen to have a deficiency of serum sodium which predisposes them to signs of agitation and irritation. Hyponatremia also affects the outcome of CHF patients. Hyponatremic CHF patients have prolonged hospital stay, a more severe form of CHF, and deranged blood pressures. As compared to normonatremic patients, hyponatremic CHF patients also have higher in-hospital mortality risk.

## Appendices

Serum Sodium Profile of Congestive Heart Failure Patients and its Impact on Their Outcome at Discharge

Patient Name: __________________________                                            MR No.: _________________________

Age: ______________________________years                                           Gender: _________________________

Date of admission: _______________________                                           Date of discharge: _________________

Body weight at admission: _______________kg                                            Height at admission: ____________cm

Residential address: ____________________________________________________________________

Contact number: ______________________________________________________________________

Co-morbidity status:

(  ) Diabetes mellitus                      (  ) Diabetic nephropathy                             (  ) Hypertension

(  ) Chronic Kidney Disease           (  ) Congestive Heart failure                        (  ) Others: _________________

Previous hospital admissions:                                          (  ) Yes                                   (  ) No

Congestive Heart failure related hospital admissions:   (  ) Yes                                   (  ) No

Treatment taken for congestive heart failure:                 (  ) Yes                                   (  ) No

Cause of current congestive heart failure:

(  ) Ischemic Heart Disease                         (  ) Hypertension                               (  ) Valvular Heart Disease

(  ) Dilated cardiomyopathy                         (  ) Restrictive cardiomyopathy        (  ) Others: _________________

Salt restricted diet:         (  ) Yes                                   (  ) No

High salt intake in diet:  (  ) Yes                                   (  ) No

Medications taking:        (  ) Furosemide                                               (  ) Spironolactone                          

                                         (  ) Hydrochlorothiazide                                 (  ) ACE Inhibitors

Clinical presentation:      (  ) Dyspnea on exertion                                (  ) Dyspnea on rest                        

                                         (  ) Orthopnea                                                 (  ) Paroxysmal nocturnal dyspnea

NYHA classification at admission:

(  ) Class I                             (  ) Class II                            (  ) Class III                           (  ) Class IV

Blood pressure at admission:

Systolic ___________mm Hg                                     Diastolic _________mm Hg

Biochemical profile at admission:

Serum sodium level: ________ mmol/L

Serum potassium level: ________ mmol/L

Serum creatinine level: ________ mg/dL

Glomerular Filtration rate: ________ mL/minute/1.73m^2^

Formula used:

GFR = 186 × (Serum Cr in mg/dL)^-1.154^ × (age in years)^-0.203^ × 0.742 (if female)

At discharge:

Date of discharge:

Duration of hospital stay:

Blood pressure at discharge:

Systolic ___________mm Hg                                     Diastolic _________mm Hg

NYHA classification at discharge:

(  ) Class I                             (  ) Class II                            (  ) Class III                           (  ) Class IV

Hospital outcome:             (  ) Alive                                (  ) Dead
